# Underdetermined DOA Estimation of Wideband LFM Signals Based on Gridless Sparse Reconstruction in the FRF Domain

**DOI:** 10.3390/s19102383

**Published:** 2019-05-24

**Authors:** Yue Cui, Junfeng Wang, Jie Qi, Zhanying Zhang, Jinqi Zhu

**Affiliations:** 1 College of Computer and Information Engineering, Tianjin Normal University, Tianjin 300387, China; zhangzhanying@tjnu.edu.cn (Z.Z.); jsjzhujinqi@tjnu.edu.cn (J.Z.); 2 School of Electrical and Electronic Engineering, Tianjin University of Technology, Tianjin 300384, China; 3 School of Electronic Science and Technology, Xiamen University, Xiamen 361005, China; qijie@xmu.edu.cn

**Keywords:** underdetermined DOA estimation, wideband LFM signals, fractional Fourier transform, sparse matrix reconstruction, atomic norm minimization

## Abstract

An underdetermined direction of arrival (DOA) estimation method of wideband linear frequency modulated (LFM) signals is proposed without grid mismatch. According to the concentration property of LFM signal in the fractional Fourier (FRF) domain, the received sparse model of wideband signals with time-variant steering vector is firstly derived based on a coprime array. Afterwards, by interpolating virtual sensors, a virtual extended uniform linear array (ULA) is constructed with more degrees of freedom, and its covariance matrix in the FRF domain is recovered by employing sparse matrix reconstruction. Meanwhile, in order to avoid the grid mismatch problem, the modified atomic norm minimization is used to retrieve the covariance matrix with the consecutive basis. Different from the existing methods that approximately assume the frequency and the steering vector of the wideband signals are time-invariant in every narrowband frequency bin, the proposed method not only can directly solve more DOAs of LFM signals than the number of physical sensors with time-variant frequency and steering vector, but also obtain higher resolution and more accurate DOA estimation performance by the gridless sparse reconstruction. Simulation results demonstrate the effectiveness of the proposed method.

## 1. Introduction

As typical wideband signals with rapidly time-varying frequency and large time-bandwidth product, linear frequency modulated (LFM) signals have been widely adopted in radar [1], sonar [2], mobile communications [3], and seismic detection fields. Direction of arrival (DOA) estimation for wideband LFM signals has played an important role in position [4], navigation and interference suppression [5] of these fields.

Due to the wideband nonstationary characteristics of the LFM signals, the traditional high-resolution DOA estimation methods based on narrowband and wide-sense stationary signals [6,7,8] cannot be applied for wideband LFM signals. The typical wideband DOA estimation methods include the incoherent signal-subspace method (ISM) [9] and the coherent signal-subspace method (CSM) [10]. The ISM decomposes the wideband signals into several independent narrowband signals by using discrete Fourier transform (DFT), and approximately assumes that the frequency is time-invariant in every frequency bin without taking the whole wideband information into account. Thus, it has a large amount of calculation and is invalid for the coherent signals. The CSM transforms the wideband signal into a certain reference frequency by the focusing matrices and takes the average of the covariance matrix to decorrelation. Although the CSM improves the accuracy of the wideband DOA estimation and has relatively low computational complexity, the focusing matrices are sensitive to a priori knowledge of the DOA which is hard to be known beforehand.

With the development of the time-frequency (TF) analysis methods, the special concentration property of wideband nonstationary signals in the TF domain can be used to distinguish from stationary signals and estimate its time-frequency signature, which motivates several approaches for the DOA estimation of wideband nonstationary signals. In [11], the DOA estimation for wideband LFM signals is realized by interpolating the spatial time–frequency distribution (STFD) matrices. Besides the disturbance of cross-terms induced by the choice of the time–frequency points, this approach mainly suffers from time consuming and model biases. To improve the performance of wideband DOA estimation, several modified algorithms based on the short-time Fourier transform (STFT) [12] and Wigner–Ville distribution (WVD) [13] have been presented. Nevertheless, the narrow time-window in STFT limits the time–frequency resolution, and the WVD requires great computational cost and suffers from cross-terms interference when nonstationary signals are multicomponent. As a linear transformation without the cross-terms interference, the fractional Fourier transform (FRFT) has no frequency point selection problem in secondary TF distribution, and can be considered as a rotation operator in the TF plane [14,15]. Moreover, owing to the excellent aggregation characteristic for the LFM signals, the FRFT is especially suitable to deal with wideband LFM signals compared with other TF analysis approaches. By using the FRFT and multiple signal classification (FRFT-MUSIC) algorithm, the DOA estimation of uncorrelated wideband LFM signals based on uniform linear array (ULA) is presented in [16], and the new received model in the fractional Fourier (FRF) domain is constructed, but it is invalid for coherent signals. The DOA estimations of coherent wideband LFM signals are proposed in [17,18] by performing a subspace smoothing and Toeplitz decorrelation scheme on the covariance matrix in FRF domain, respectively, at the cost of a reduction in array aperture.

Meanwhile, compressive sensing (CS) and sparse representation (SR) have been rapidly developed, and the spatial sparsity has been introduced in the DOA estimation of wideband signals. In [19,20], using the idea of the ISM or the CSM methods, the wideband signals are decomposed or transformed into narrowband signals, and then the spatial sparse solution of DOA is obtained by using the CS algorithms, such as orthogonal matching pursuit (OMP) [19] or singular value decomposition ($l_{1}$-SVD) reconstruction [20]. Motivated by the FRFT-MUSIC algorithm, the received model of wideband signals in the FRF domain is in accordance with the CS framework, and the spatial sparse solution of DOA can be recovered under the maximizing a posteriori (MAP) criterion based on $l_{p}$ norm [21]. In [22], a spatial compressed sensing framework is employed for DOA estimation with a randomly thinned phased array. The above methods based on the CS framework are also applicable to correlated sources, even with a single snapshot. However, there is an inherent disadvantage in these methods, i.e., the grid mismatch problem, which is inevitable to bring substantial bias especially when the DOAs of signals deviate from the discrete grids or SNR increases. Their performance would be compromised with the grid sparsity in the CS methods. Furthermore, the aforementioned CS-based and TF-based algorithms are all based on ULA or random array, so the degrees of freedom (DOFs) are limited by the number of physical sensors, i.e., the number of wideband signals identified by these methods should be less than the number of physical sensors in the array.

In order to increase DOFs and address the scenario where the number of signal sources is more than that of physical sensors, the nested arrays [23] and the coprime arrays [24,25,26,27,28,29], as two typical sparse arrays, have been used in the underdetermined DOA estimation algorithms. In [24,25,26,27,28], by adopting a coprime array as the received array of narrowband signals, a virtual extended array is constructed by vectoring the covariance matrix, which is also called the difference co-array. Although the difference co-array has some holes, which make it discontiguous, there are more virtual sensors in it than the physical sensors in a coprime array. In [26,27], discrete grids are defined at the spatial directions and the CS framework is used in the difference co-array, so the underdetermined DOA estimation of a narrowband signal is transformed to a spatial sparse recovery problem based on the CS framework, and many CS recovery algorithms, such as the least absolute shrinkage and selection operator (LASSO) [26] and the OMP [27], can be adopted. Nevertheless, the grid mismatch problem still exists due to the usage of the CS algorithms. In [29], by extending the difference co-array to wideband cases, a two-step off-grid group sparsity approach for wideband DOA estimation is proposed to solve the underdetermined and grid mismatch problem. It firstly yields a coarser grid estimation, and then a bias vector is estimated by the optimization. However, it still uses the idea of ISM with time-invariant frequency and steering vector in every frequency bin, and mismatches with the wideband LFM signals model, which has the contiguous and linearly time-varying frequency and the time-variant steering vector. Moreover, the off-grid DOA estimation is still based on the discrete grids of spatial parameters, so the recovery deviation is only improved to a certain extent.

Recently, the grid-free compressed sensing approach based on a continuous dictionary has been proposed by atomic norm minimization (ANM) [30], which can recover continuous-valued frequencies of the spectrally sparse signal from a few time-domain samples. Authors of [31] modified the ANM to recover super-resolution frequencies with the prior knowledge from the structure of a spectrally sparse and undersampled signal. In [32], an iterative reweighted ANM algorithm is proposed to improve frequency recovery performance with faster speed. Although these methods are applied to recover continuous-valued frequencies in spectral estimation, they provide an effective approach to solve the discrete grid mismatch problem for non-uniformly sampled or irregularly spaced signals.

The focus of the proposed method is to solve the underdetermined DOA estimation of wideband LFM signals with gridless sparse reconstruction. By using the concentration property of wideband LFM signals in the FRF domain, the received sparse model in the FRF domain is firstly constructed based on the coprime array. Then, by interpolating virtual sensors into a difference co-array, the sparse covariance matrix of virtual ULA is recovered by modified atomic norm minimization, which increases the DOFs and provides a contiguous basis to avoid discrete grids mismatch. Unlike some existing algorithms that assume the steering vector of wideband signals is time-invariant in every frequency bin, the proposed method not only can resolve more wideband LFM sources than physical sensors with time-variant steering vector in FRF domain, but can also obtain more accurate DOA estimation performance with gridless sparse reconstruction.

Notations: The superscripts ${\left(\cdot \right)}^{T}$, ${\left(\cdot \right)}^{*}$, ${\left(\cdot \right)}^{H}$ and ${\left(\cdot \right)}^{-1}$ respectively represent the transpose, conjugation, conjugate transpose and inverse of a matrix. Let the operator rank (·) and Tr (·) indicate the rank and the trace of a matrix, respectively. The notation ⊗ and ⊙ respectively signify the Kronecker product and Hadamard product between matrices. ${∥\cdot ∥}_{F}$ and |·| mean the Frobenius norm and cardinality of a set, respectively.

## 2. The Received Model of Wideband LFM Signal

**Figure 1:**
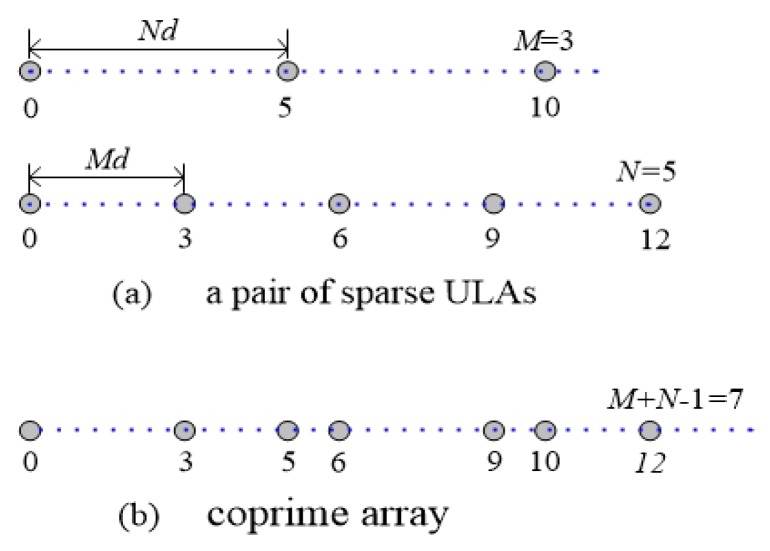
The structure of coprime array with M=3 and N=5.

Consider there is a pair of sparse ULAs with *M* and *N* isotropic sensors, respectively, where *M* and *N* are coprime integers. As shown in Figure 1a, align the first sensor of both sparse ULAs as the reference, and keep the inter-element distances of the two sparse ULAs with *Md* and *Nd*, respectively, where *d* denotes the half of wavelength. According to the coprimality, *M + N* − 1 sensors constitute a coprime array without overlap which can be seen in Figure 1b, and the *i*th sensor locates at $q_{i}d$, where i=1,2,⋯,M+N−1 and $q_{i}\in Q=\left\{Mn\right\}\cup \left\{Nm\right\}$, 0 ≤n≤N−1, 0 ≤m≤M−1.

### 2.1. The Received Signal Model in Time Domain

Without loss of generality, assume there are *K* wideband LFM signals from different directions $\theta_{k},k=1,2,\cdots ,K$ impinging on the coprime array. The output of the *i*th sensor can be described as

(1) $$x_{i}\left(t\right)=\sum_{k=1}^{K}s_{k}\left(t-\tau_{k,i}\right)+n_{i}\left(t\right),$$

where $s_{k}\left(t\right)=e^{j2\pi (f_{k}t+\frac{1}{2}\mu_{k}t^{2})}$ is the *k*th wideband LFM signal, $\tau_{k,i}=q_{i}d\sin\theta_{k}/c$ is the time delay of *k*th signal at *i*th senor, $f_{k}$ and $\mu_{k}$ are respectively its initial frequency and frequency modulation rate, *c* is light velocity, i=1,2,⋯,M+N−1, and k=1,2,⋯,K. The received signals based on coprime array can be written as

(2) $$\begin{array}{cc} x(t) & ={\left[x_{1}\left(t\right),x_{2}\left(t\right),\cdots ,x_{M+N-1}\left(t\right)\right]}^{T}\\ & =\sum_{k=1}^{K}s_{k}\left(t-\tau_{k}\right)+n\left(t\right)\\ & =\sum_{k=1}^{K}a\left(\theta_{k},t\right)s_{k}\left(t\right)+n\left(t\right)\\ & =\mathrm{As}(t)+n(t), \end{array}$$

where $s_{k}\left(t\right)$ and $a(\theta_{k},t)$ are respectively the *k*th wideband LFM signal and its steering vector, n(t) is the additive white Gaussian noise vector which is statistically independent of signals, and where, for simplicity, $s\left(t\right)={\left[s_{1}\left(t\right),\cdots ,s_{K}\left(t\right)\right]}^{T}$ and $A=[a\left(\theta_{1},t\right),\cdots ,a\left(\theta_{K},t\right)]$ denote the signal vector and the array manifold, respectively. The steering vector $a(\theta_{k},t)$ of the *k*th wideband LFM signal is given by

(3) $$\begin{array}{cc} a(\theta_{k},t) & ={\left[1,e^{-j2\pi \left(f_{k}+\mu_{k}t\right)\tau_{k,2}+j\pi \mu_{k}{\left(\tau_{k,2}\right)}^{2}},\cdots ,e^{-j2\pi \left(f_{k}+\mu_{k}t\right)\tau_{k,(M+N-1)}+j\pi \mu_{k}{\left(\tau_{k,(M+N-1)}\right)}^{2}}\right]}^{T}\\ & ={\left[1,e^{-j2\pi f_{k}\left(t\right)\tau_{k,2}}e^{j\pi \mu_{k}{\left(\tau_{k,2}\right)}^{2}},\cdots ,e^{-j2\pi f_{k}\left(t\right)\tau_{k,(M+N-1)}}e^{j\pi \mu_{k}{\left(\tau_{k,(M+N-1)}\right)}^{2}}\right]}^{T}, \end{array}$$

where $f_{k}\left(t\right)=f_{k}+\mu_{k}t$ and $\tau_{k,i}=q_{i}d\sin\theta_{k}/c$. We can clearly observe that $f_{k}\left(t\right)$ and $a(\theta_{k},t)$ of the *k*th wideband LFM signal both change with time. It should be noted that $a(\theta_{k},t)$ is not only relevant to DOAs of signals, but also varies with time. Since the frequency and steering vector of the LFM signal are time-variant, the DOA estimation methods based on narrow and stationary signals which assume the time-invariant frequency and steering vector in [6,7,8] cannot be applied to resolve wideband signals in the receiver. Moreover, the traditional wideband DOA estimation methods, such as ISM-based methods [9,19,20,28] approximately assume that the frequency and steering vector of wideband signals are time-invariant in every frequency bin when the received signals are modeled, are different from the model of the proposed method in Equation (2). The proposed method considers the scenarios where frequencies of wideband signal continuously and linearly change with time, and is suitable for the DOA estimation of wideband LFM signal.

### 2.2. FRFT of Wideband LFM Signal

As a generalization of Fourier transform (FT), FRFT is regarded as a counterclockwise rotation of the signal coordinates around the origin in the TF plane. The definition of FRFT for the *k*th LFM signal $s_{k}\left(t\right)$ is written as

(4) $$\begin{array}{c} \;S_{k}\left(\alpha ,u\right)=F^{p}\left[s_{k}\left(t\right)\right]=\int_{-\infty }^{+\infty }K_{p}\left(u,t\right)s_{k}\left(t\right)\;dt \end{array}$$

where *p* presents the order of FRFT, α=pπ/2 denotes a counterclockwise rotation angle of the signal coordinates and *u* stands for the FRF domain. $K_{p}\left(u,t\right)$ means the FRFT kernel function, i.e.,

(5) $$K_{p}\left(u,t\right)=\left\{\begin{array}{c} \sqrt{1-j\cot\alpha }e^{j\pi [\left(t^{2}+u^{2}\right)\cot\alpha -2tu\csc\alpha ]},\alpha \neq n\pi \\ \delta (t-u),\;\;\;\alpha =2n\pi \\ \delta (t+u),\;\;\;\alpha =(2n+1)\pi . \end{array}\right.$$

From the above definition in Equations (4) and (5), we can easily find that the FRFT is a linear transformation without the cross-terms interference especially when $s_{k}\left(t\right)$ includes multicomponents. The inverse FRFT can be expressed as

(6) $$s_{k}\left(t\right)=F^{-p}\left[S_{k}\left(\alpha ,u\right)\right]=\int_{-\infty }^{\infty }K_{-p}\left(u,t\right)S_{k}\left(\alpha ,u\right)\;du.$$

**Figure 2:**
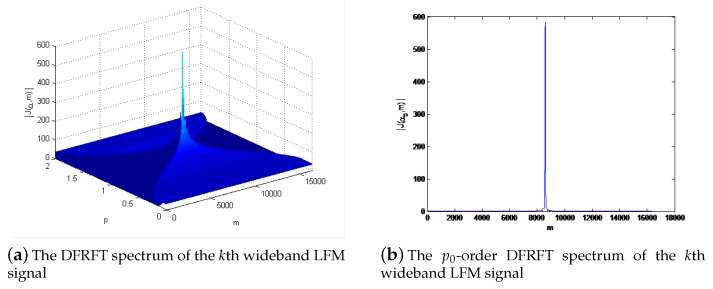
The spectrum for the DFRFT of the *k*th wideband LFM signal.

It reveals that $s_{k}\left(t\right)$ can be decomposed to a basis formed by the orthonormal LFM functions in the FRF domain, which is especially appropriate to process the LFM signals. Furthermore, an LFM signal can be transformed into an impulse δ-function in a certain fractional domain, which would generate an obvious peak in the FRF domain as shown in Figure 2. Therefore, as for the LFM signal, the best aggregation performance can be obtained by FRFT in a certain FRF domain [14].

According to the decomposition algorithm proposed by Ozaktas [15], $s_{k}\left(t\right)$ is sampled into snapshots $s_{k}\left(n\right)$ at the sampling rate $f_{s}$, and the discrete FRFT (DFRFT) of $s_{k}\left(n\right)$ can be expressed as

(7) $$\begin{array}{cc} S_{k}\left(\alpha ,m\right) & =\sum_{n=-(P-1)/2}^{(P-1)/2}K_{p}\left(m,n\right)s_{k}\left(n\right)=\sqrt{\frac{1-j\cot\alpha }{P}}e^{j\pi (\frac{m^{2}\cot\alpha }{P})}\cdot \sum_{n=-(P-1)/2}^{(P-1)/2}e^{j\pi n(\frac{-2m\csc\alpha }{P}+\frac{2f_{k}}{f_{s}})}e^{j\pi n^{2}\left(\frac{\cot\alpha }{P}+\frac{\mu_{k}}{f_{s}^{2}}\right)}, \end{array}$$

where *P* is the number of snapshots and *m* denotes the quantification of *u* in the FRF domain, which can be seen in Figure 2. According to the concentration property of LFM signal in FRF domain, it has been proved $S_{k}\left(\alpha ,m\right)$ has the best concentration at $\alpha_{k0}$ = $-\cot^{-1}\left(\frac{\mu_{k}P}{f_{s}^{2}}\right)$, i.e.,

(8) $$S_{k}\left(\alpha_{k0},m\right)=\sqrt{\frac{1-j\cot\alpha_{k0}}{P}}e^{j\pi (\frac{m^{2}\cot\alpha_{k0}}{P})}\cdot \sum_{n=-(P-1)/2}^{(P-1)/2}e^{j2\pi n(\frac{-m\csc\alpha_{k0}}{P}+\frac{f_{k}}{f_{s}})}.$$

It is obvious that when $m_{k0}=\frac{f_{k}P\sin\alpha_{k0}}{f_{s}}$, $S_{k}\left(\alpha_{k0},m\right)$ reaches the maximum value

(9) $$S_{k}\left(\alpha_{k0},m_{k0}\right)=\sqrt{P(1-j\cot\alpha_{k0})}e^{j\pi (\frac{m_{k0}^{2}\cot\alpha_{k0}}{P})}.$$

At this moment, the parameters $\left(\mu_{k},f_{k}\right)$ of the *k*th LFM signal can be estimated by the coordinates $\left(\alpha_{k0},m_{k0}\right)$ corresponding to the peak $S_{k}\left(\alpha_{k0},m_{k0}\right)$ [16]

(10) $$\left\{\begin{array}{c} \;{\hat{\mu }}_{k}=-f_{s}^{2}\cot\alpha_{k0}/P\\ \;{\hat{f}}_{k}=m_{k0}f_{s}\csc\alpha_{k0}/P. \end{array}\right.$$

### 2.3. The Received Signal Model of Wideband LFM Signal Based on Coprime Array in FRF Domain

The *k*th LFM signal received by the *i*th sensor can be expressed as

(11) $$\begin{array}{cc} s_{k,i}\left(t\right) & =s_{k}\left(t-\tau_{k,i}\right)\\ & =e^{j2\pi (-f_{k}\tau_{k,i}+\frac{1}{2}\mu_{k}\tau_{k,i}^{2})}\cdot e^{j2\pi (\left(f_{k}-\mu_{k}\tau_{k,i}\right)t+\frac{1}{2}\mu_{k}t^{2})}\\ & =b_{k}\left(\tau_{k,i}\right)\cdot e^{j2\pi (f_{k}^{\prime }t+\frac{1}{2}\mu_{k}t^{2})}, \end{array}$$

where $b_{k}\left(\tau_{k,i}\right)=e^{j2\pi (-f_{k}\tau_{k,i}+\frac{1}{2}\mu_{k}\tau_{k,i}^{2})}$ and $f_{k}^{\prime }=f_{k}-\mu_{k}\tau_{k,i}$. Compared with the signal $s_{k}\left(t\right)$ received by the reference sensor, the phase and initial frequency of $s_{k,i}\left(t\right)$ both change, and only its frequency modulation rate $\mu_{k}$ remains the same. Combined with Equation (10), we can deduce that the DFRFT $S_{k,i}\left(\alpha ,m\right)$ remains the best aggregation at $\alpha_{k0}$. It can be derived that the coordinates and amplitude corresponding to the peak of $S_{k,i}\left(\alpha_{k0},m\right)$ are changed into $\left(\alpha_{k0},m_{k,i}\right)$ and $S_{k,i}\left(\alpha_{k0},m_{k,i}\right)$, where

(12) $$\left\{\begin{array}{c} \;\alpha_{k0}=-\mathrm{arc}\cot\left(\mu_{k}P/f_{s}^{2}\right)\\ \;m_{k,i}=m_{k0}+f_{s}\tau_{k,i}\cos\alpha_{k0} \end{array}\right.$$

(13) $$\begin{array}{cc} S_{k,i}\left(\alpha_{k0},m_{k,i}\right) & =S_{k}\left(\alpha_{k0},m_{k0}\right)\cdot e^{-j2\pi f_{s}\tau_{k,i}m_{k0}\sin\alpha_{k0}/P}\cdot e^{-j\pi f_{s}^{2}\tau_{k,i}^{2}\sin\alpha_{k0}\cos\alpha_{k0}/P}\\ & =S_{k}\left(\alpha_{k0},m_{k0}\right)\cdot c_{k}\left(\tau_{k,i}\right), \end{array}$$

where $c_{k}\left(\tau_{k,i}\right)=e^{-j2\pi f_{s}\tau_{k,i}m_{k0}\sin\alpha_{k0}/P}e^{-j\pi f_{s}^{2}\tau_{k,i}^{2}\sin\alpha_{k0}\cos\alpha_{k0}/P}$. Meanwhile, according to the time-shifting characteristics of FRFT [14], the same deduction as Equation (13) also can be proved again. It is noteworthy that the quadratic term about time delay $\tau_{k,i}^{2}$ in Equation (13) is so small that it can be ignored in processing [5,16,17,18,33]. Hence, $c_{k}\left(\tau_{k,i}\right)$ can be approximated as

(14) $$c_{k}\left(\tau_{k,i}\right)=e^{-j2\pi f_{s}\tau_{k,i}m_{k0}\sin\alpha_{k0}/P}.$$

According to the aforementioned analysis, the peaks of received signals based on coprime array in the FRF domain can be expressed as

(15) $$\begin{array}{cc} X_{F} & ={\left[X_{1},X_{2},\cdots ,X_{M+N-1}\right]}^{T}\\ & =C\cdot S_{F}+N_{F}, \end{array}$$

where $S_{F}$ and $N_{F}$ are the *k*th wideband LFM signal and noise components of the peak of received signals in the FRF domain, respectively, and where

(16) $$\begin{array}{c} S_{F}=diag\left(S_{1}\left(\alpha_{10},m_{10}\right),S_{2}\left(\alpha_{20},m_{20}\right),\cdots ,S_{K}\left(\alpha_{K0},m_{K0}\right)\right), \end{array}$$

(17) $$\begin{array}{c} \begin{array}{cc} C & ={\left[c_{1},c_{2},\cdots ,c_{K}\right]}_{(M+N-1)\times K}=\left[\begin{array}{cccc} 1 & 1 & \cdots & 1\\ c_{1}\left(\tau_{1,2}\right) & c_{2}\left(\tau_{2,2}\right) & \cdots & c_{K}\left(\tau_{K,2}\right)\\ \vdots & \vdots & \ddots & \vdots \\ c_{1}\left(\tau_{1,M+N-1}\right) & c_{2}\left(\tau_{2,M+N-1}\right) & \cdots & c_{K}\left(\tau_{K,M+N-1}\right) \end{array}\right], \end{array} \end{array}$$

where diag(·) means a diagonal matrix and $c_{k}={\left[1,c_{k}\left(\tau_{k,2}\right),\cdots ,c_{k}\left(\tau_{k,M+N-1}\right)\right]}^{T}$ denotes the steering vector of the *k*th LFM signal in the FRF domain. From Equation (14), it can be clearly seen that $c_{k}$ is a time-invariant steering vector and only varies with $\theta_{k}$. The covariance matrix of $X_{F}$ is given by

(18) $$\begin{array}{cc} R_{X_{F}} & =E\left\{X_{F}X_{F}^{H}\right\}\\ & =\sum_{k=1}^{K}p_{k}c_{k}\left(\theta_{k}\right)c_{k}^{H}\left(\theta_{k}\right)+\sigma_{n}^{2}I\\ & =CR_{S}C^{H}+R_{N}, \end{array}$$

where E{·} denotes the statistical expectation, $R_{S}$ and $R_{N}$ are respectively the covariance matrices of $S_{F}$ and $N_{F}$, I denotes the identity matrix and $p_{k}=E\left[|S_{k}\left(\alpha_{k0},m_{k0}\right){|}^{2}\right]$ denotes the second-order statistics of peak $S_{k}\left(\alpha_{k0},m_{k0}\right)$ of the *k*th LFM signal in the FRF domain.

## 3. Covariance Matrix Reconstruction by Using Atomic Norm Based on Difference Co-Array Interpolation

### 3.1. Interpolating Virtual Sensors in the Difference Co-Array to Form the Virtual ULA

By vectoring the covariance matrix $R_{X_{F}}$, the virtual received signal can be expressed as

(19) $$\begin{array}{cc} Y_{F} & =vec(R_{X_{F}})\\ & =vec(\sum_{k=1}^{K}p_{k}c_{k}\left(\theta_{k}\right)c_{k}^{H}\left(\theta_{k}\right)+\sigma_{n}^{2}I)\\ & =\mathrm{Dp}+\sigma_{n}^{2}i, \end{array}$$

where vec(·) is the vectorization operator, D=$[c_{1}^{*}\left(\theta_{1}\right)⊗c_{1}\left(\theta_{1}\right)\;c_{2}^{*}\left(\theta_{2}\right)⊗c_{2}\left(\theta_{2}\right)\cdots \;c_{K}^{*}\left(\theta_{K}\right)⊗c_{K}\left(\theta_{K}\right)]$, $p={\left[p_{1},p_{2},\cdots ,p_{K}\right]}^{T}$ and i=vec(I). The dimensions of the virtual received signal $Y_{F}$ are increased to ${\left(M+N-1\right)}^{2}\times 1$. Compared with Equation (15), we found that $Y_{F}$ behaves in a similar linear structure as Equation (15), whose manifold D plays the same role as the manifold of a virtual array. The sensors corresponding to manifold D are located at the set $\left\{s_{k}d\right\}$, where

(20) $$S=\left\{s_{k}\right\}=\left\{q_{i}-q_{j}\left|i,j=0,1,\cdots ,M+N-1\right.\right\}.$$

**Figure 3:**
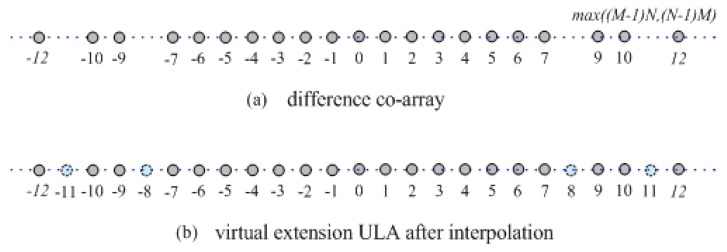
The structure of virtual extension arrays based on coprime array with M=3 and N=5.

By extracting the different elements and removing the repetitive elements from S, a subset $S_{v}=\left\{v_{k}\right\}$ is built. And then, a difference co-array $A_{v}$ is derived with the sensors located at $S_{v}d=\left\{v_{k}d\right\}$, whose configuration is shown in Figure 3a. Correspondingly, the virtual received signals $Y_{v}$ of the difference co-array $A_{v}$ can be obtained by selecting the corresponding rows from $Y_{F}$ as follows

(21) $$Y_{v}=D_{v}p+\sigma_{n}^{2}\tilde{I},$$

where $D_{v}\in C^{\left|S_{v}\right|\times K}$ denotes the array manifold of the difference co-array $A_{v}$. As demonstrated in [25], the set $S_{v}$ ranges from −M(N−1) to M(N−1), but it is not continuous. Compared with ULA, there are several holes in $A_{v}$ as shown in Figure 3a. Since the non-uniformity and sparsity of an array result in the model mismatch by using the traditional DOA estimation methods, Ref. [25] selects the consecutive part $S_{c}$ in $A_{v}$ to avoid this problem, but the information of the discontiguous part in $A_{v}$ is apparently discarded, which inevitably degrades the estimation performance as analyzed in [24,26].

In order to make the most of all sensors in the difference co-array $A_{v}$, by interpolating virtual sensors at discontiguous position, the missing sensors at holes of $A_{v}$ are added on. Then, a virtual longer ULA $A_{U}$ is built with 2M(N−1)+1 sensors located at $S_{U}d=\left\{[-M(N-1),\cdots ,-1,0,1,\cdots ,M(N-1)]d\right\}$, which is shown in Figure 3b. It should be noted that the received signals corresponding to the interpolated virtual sensors are set to zero, which are only the virtual signals in the mathematical rather than physical sense. Therefore, the 2M(N−1)+1 dimensional virtual received signals $Y_{U}$ for virtual ULA $A_{U}$ can be defined as

(22) $${\langle Y_{U}\rangle }_{i}=\left\{\begin{array}{c} \;{\langle Y_{v}\rangle }_{i},i\in S_{v}\\ \;0,\;\;i\in S_{U}-S_{v}, \end{array}\right.$$

where ${\langle \cdot \rangle }_{i}$ means the signal received by the *i*th sensor of virtual ULA $A_{U}$. If we can recover the unknown virtual signals corresponding to the interpolated virtual sensors by sparse signal processing, the reconstructed received signals corresponding to the longer virtual ULA $A_{U}$ can be applied by the traditional DOA estimation methods in order to improve the accuracy and DOFs.

### 3.2. Sparse Covariance Matrix Reconstruction by Atomic Norm Minimization

Assume the signals received by the interpolated virtual sensors are actually existed rather than the nominal as in Equation (22), but they are unknow. Then, the signals received by the virtual ULA $A_{U}$ can be expressed as

(23) $$Y=\sum_{k=1}^{K}u\left(\theta_{k}\right)p\left(\theta_{k}\right)=\mathrm{Up},$$

where $U=\left[u\left(\theta_{1}\right),u\left(\theta_{2}\right),\cdots ,u\left(\theta_{K}\right)\right]\in C^{\left|S_{U}\right|\times K}$ represents the array manifold of virtual ULA $A_{U}$. Seen from the outside, the received signal Y in Equation (23) also has a similar linear structure as the received signal $X_{F}$ on coprime array without noise, but Y actually includes a second-order statistics derived from the covariance matrix of signal peaks in FRF domain Equation (18). Furthermore, Y is a column vector and has similar characteristic as a single snapshot, so the low-rank problem of second-order statistics in Y would bring difficulty to estimate DOAs of multiple sources.

**Figure 4:**
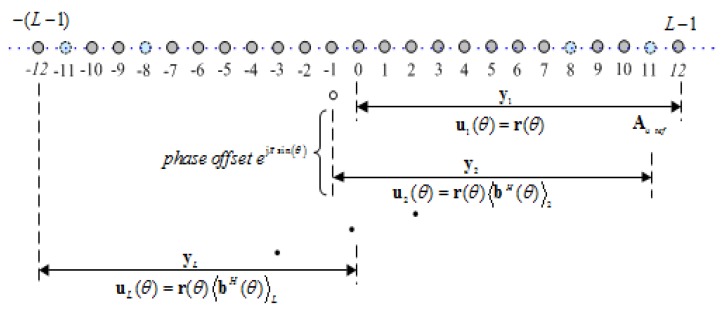
The division of sub-array in virtual ULA $S_{U}$ with M=3 and N=5.

On account of the low-rank problem, taking the middle sensor as the reference, the virtual ULA $S_{U}$ can be divided into L=M(N−1) + 1 overlapping sub-arrays, and each sub-array includes *L* contiguous virtual sensors, which is shown in Figure 4. Accordingly, Y can be divided into *L* sub-vectors $\left\{y_{1},y_{2},\cdots ,y_{L}\right\}$.

The first sub-array could be taken as the reference virtual sub-array, and its steering vector can be written as

(24) $$\begin{array}{cc} r(\theta_{k}) & =u_{1}\left(\theta_{k}\right)\\ & ={\left[e^{-j2\pi f_{s}\tau_{k,L}\left(m_{k0}\sin\alpha_{k0}\right)/P},e^{-j2\pi f_{s}\tau_{k,L+1}\left(m_{k0}\sin\alpha_{k0}\right)/P},\cdots ,e^{-j2\pi f_{s}\tau_{k,2L-1}\left(m_{k0}\sin\alpha_{k0}\right)/P}\right]}^{T}\\ & ={\left[1,e^{-j2\pi f_{s}d\sin\left(\theta_{k}\right)\left(m_{k0}\sin\alpha_{k0}\right)/Pc},\cdots ,e^{-j2\pi f_{s}\left(L-1\right)d\sin\left(\theta_{k}\right)\left(m_{k0}\sin\alpha_{k0}\right)/Pc}\right]}^{T}. \end{array}$$

Likewise, the steering vector of the *k*th signal received by the *l*th virtual sub-array is

(25) $$\begin{array}{cc} u_{l}\left(\theta_{k}\right) & ={\left[e^{-j2\pi f_{s}\tau_{k,L-l+1}\left(m_{k0}\sin\alpha_{k0}\right)/P},e^{-j2\pi f_{s}\tau_{k,L-l+2}\left(m_{k0}\sin\alpha_{k0}\right)/P},\cdots ,e^{-j2\pi f_{s}\tau_{k,2L-l}\left(m_{k0}\sin\alpha_{k0}\right)/P}\right]}^{T}\\ & ={\left[e^{-j2\pi f_{s}\left(-l+1\right)d\sin\theta_{k}\left(m_{k0}\sin\alpha_{k0}\right)/Pc},e^{-j2\pi f_{s}\left(-l+2\right)d\sin\theta_{k}\left(m_{k0}\sin\alpha_{k0}\right)/Pc},\cdots ,e^{-j2\pi f_{s}\left(-l+L\right)d\sin\theta_{k}\left(m_{k0}\sin\alpha_{k0}\right)/Pc}\right]}^{T}, \end{array}$$

where $\tau_{k,L-l+i}=\left(-l+i\right)d\sin\theta_{k}/c$ denotes the time delay of the *k*th signal at the *i*th sensor in the *l*th virtual sub-array. Hence, the signal received by the *l*th virtual sub-array is expressed as

(26) $$y_{l}=\sum_{k=1}^{K}u_{l}\left(\theta_{k}\right)p_{k}=U_{l}p,\;l=1,2,\cdots ,L$$

where $U_{l}=\left[u_{l}\left(\theta_{1}\right),u_{l}\left(\theta_{2}\right),\cdots ,u_{l}\left(\theta_{K}\right)\right]\in C^{L\times K}$. Note that the virtual received signal $y_{l}$ by the *l*th virtual sub-array denotes the second-order statistics of peak $S_{k}\left(\alpha_{k0},m_{k0}\right)$, unlike the received model in Equation (15) which contains the peak $S_{k}\left(\alpha_{k0},m_{k0}\right)$ of the *k*th wideband LFM signal in FRF domain. The division of the *L* sub-arrays would result in the phase offsets among virtual received signal $y_{l}$. The phase offsets between reference virtual sub-array and the *L*-1 sub-arrays can be deduced as

(27) $$\begin{array}{cc} b(\theta ) & ={\left[1,e^{-j2\pi f_{s}d\sin\left(\theta \right)\left(m_{0}\sin\alpha_{0}\right)/Pc},\cdots ,e^{-j2\pi f_{s}\left(L-1\right)d\sin\left(\theta \right)\left(m_{0}\sin\alpha_{0}\right)/Pc}\right]}^{T}, \end{array}$$

which represents the difference among each virtual sub-array. From the above analysis, the steering vector and received signal of the *l*th sub-array also can be respectively expressed as

(28) $$u_{l}\left(\theta \right)=u_{1}\left(\theta \right){\langle b^{H}\left(\theta \right)\rangle }_{l}=r\left(\theta \right){\langle b^{H}\left(\theta \right)\rangle }_{l}$$

and

(29) $$\begin{array}{cc} y_{l} & =\sum_{k=1}^{K}u_{l}\left(\theta_{k}\right)p_{k}=\sum_{k=1}^{K}r\left(\theta_{k}\right){\langle b^{H}\left(\theta_{k}\right)\rangle }_{l}p_{k}=r\cdot diag\left(b_{l}^{H}\right)p. \end{array}$$

By aligning the *L* sub-vectors as follows

(30) $$\tilde{Y}=\left[y_{l},y_{2},\cdots ,y_{L}\right]\in C^{L\times L},$$

a virtual received signal model is constructed. Based on the deduction of Equation (29), $\tilde{Y}$ is regarded as *L* different measurements of the virtual signals received by the reference virtual sub-array, but it contains all elements in Y, so it includes all the information in the virtual ULA $A_{U}$. Hence, $\tilde{Y}$ is used to estimate DOA instead of Y, which not only solves the low-rank problem of Y, but also retains all the elements in Y to improve the estimation performance.

In the sparse recovery, as a continuous representation method without discrete basis and grid mismatch, atomic norm is used to exactly reconstruct signal. In order to recover $\tilde{Y}$, the definition of the atomic norm about $\tilde{Y}$ is introduced as follows

(31) $$\begin{array}{c} {∥\begin{array}{c} \tilde{Y} \end{array}∥}_{A,0}=\underset{K}{\inf}\left\{\sum_{k=1}^{K}r\left(\theta_{k}\right)b^{H}\left(\theta_{k}\right)p_{k},p_{k}\geq 0\right\}=\underset{K}{\inf}\left\{\sum_{k=1}^{K}B\left(\theta_{k}\right)p_{k},p_{k}\geq 0\right\}, \end{array}$$

where $A=\left\{B(\theta )\right\}=\left\{r\left(\theta \right)b^{H}\left(\theta \right)|\theta \in \left[-90^{\circ },90^{\circ }\right]\right\}$ is the atomic set, and the spatial parameter θ varies continuously. Using the least atoms to represent $\tilde{Y}$ is an NP-hard problem, which is equivalent to minimize Equation (31). It can be relaxed to the convex optimization problem

(32) $$\begin{array}{c} {∥\begin{array}{c} \tilde{Y} \end{array}∥}_{A}=\inf\left\{\sum_{k}p_{k}|\tilde{Y}=\sum_{k}B\left(\theta_{k}\right)p_{k},p_{k}\geq 0\right\}, \end{array}$$

and can be further represented as a semi-definite programming (SDP) form [30] as follows

(33) $$\begin{array}{c} {∥\begin{array}{c} \tilde{Y} \end{array}∥}_{A}=\underset{\begin{array}{c} z\in C^{L},W\in C^{L\times L} \end{array}}{\inf}\left\{\frac{1}{2L}\mathrm{Tr}\left(T\left(z\right)\right)+\frac{1}{2L}\mathrm{Tr}\left(W\right)|\left[\begin{array}{cc} T(z) & \tilde{Y}\\ {\tilde{Y}}^{H} & W \end{array}\right]\geq 0\right\}, \end{array}$$

where T(z) denotes a Hermitian Toeplitz matrix and z is its first column. Combined with Vandermonde decomposition [34], T(z) can be denoted as

(34) $$T\left(z\right)=\sum_{k=1}^{K}r\left(\theta_{k}\right)r^{H}\left(\theta_{k}\right)p_{k},$$

where $p_{k}=E\left[|S_{k}\left(\alpha_{k0},m_{k0}\right){|}^{2}\right]$ denotes the second-order statistics of the *k*th LFM signal peak $S_{k}\left(\alpha_{k0},m_{k0}\right)$ in FRF domain. If $z^{*}$ is the optimum solution for Equation (33), $T(z^{*})$ equals to the covariance matrix of $S_{k}\left(\alpha_{k0},m_{k0}\right)$ in the FRF domain received by the reference virtual array.

Compared Equation (24) with Equation (27), it is obvious that r(θ)=b(θ). And then combined with Equations (29) and (34) can be further expressed as

(35) $$\begin{array}{cc} T(z^{*}) & =\sum_{k=1}^{K}r\left(\theta_{k}\right)b^{H}\left(\theta_{k}\right)p_{k}=\tilde{Y}, \end{array}$$

where $z^{*}$ denotes the optimum solution to Equation (33). Meanwhile, the first column $z^{*}$ of the Hermitian Toeplitz positive semidefinite (PSD) matrix $T(z^{*})$ also can be expressed as

(36) $$\begin{array}{cc} z^{*} & =\sum_{k=1}^{K}r\left(\theta_{k}\right){\langle b^{H}\left(\theta_{k}\right)\rangle }_{1}p_{k}=\sum_{k=1}^{K}u_{1}\left(\theta_{k}\right)p_{k}=y_{1}, \end{array}$$

i.e., the first column of $\tilde{Y}$. If we can retrieve $z^{*}$ by atomic norm, $T(z^{*})$ can be reconstructed based on the Hermitian Toeplitz property. That is also to say $\tilde{Y}$ and covariance matrix of reference virtual sub-array are acquired, which could be used to precisely estimate DOA by traditional methods. Due to the continuous direction parameter by atomic norm, there is no discrete grid mismatch problem. Thus, focus of the following work is to recover the first column $z^{*}$.

As derivation in Equation (36), we can get the atomic norm of z as follows

(37) $${∥\begin{array}{c} z \end{array}∥}_{A_{r}}=\inf\left\{\sum_{k}p_{k}|z=\sum_{k}r\left(\theta_{k}\right)p_{k},p_{k}\geq 0\right\},$$

where $A_{r}=\left\{r\left(\theta \right)=u_{1}\left(\theta \right)|\theta \in \left[-90^{\circ },90^{\circ }\right]\right\}$ is the atomic set of z with continuous direction parameter. Note that compared ${∥\begin{array}{c} z \end{array}∥}_{A_{r}}$ in Equation (37) with ${∥\begin{array}{c} \tilde{Y} \end{array}∥}_{A}$ in Equation (32), the atom set $A_{r}$ of z is the same as the atom set A of $\tilde{Y}$, and minimizing ${∥\begin{array}{c} \tilde{Y} \end{array}∥}_{A}$ is equivalent to minimizing ${∥\begin{array}{c} z \end{array}∥}_{A_{r}}$ because of ${\langle b^{H}\left(\theta_{k}\right)\rangle }_{1}=1$. Therefore, the reconstruction of $\tilde{Y}$, i.e., the covariance matrix $T(z^{*})$ on reference virtual array, can be achieved by minimizing ${∥\begin{array}{c} z \end{array}∥}_{A_{r}}$.

In actual application, according to Equations (35) and (30), the covariance matrix of received signal peaks on the reference virtual array in the FRF domain can be obtained by virtual received signals $Y_{U}$ as

(38) $$\begin{array}{cc} \tilde{R} & =T\left(y_{1}\right)=\left[\begin{array}{cccc} {\langle Y_{U}\rangle }_{L} & {\langle Y_{U}\rangle }_{L+1}^{*} & \cdots & {\langle Y_{U}\rangle }_{2L-1}^{*}\\ {\langle Y_{U}\rangle }_{L+1} & {\langle Y_{U}\rangle }_{L} & \cdots & {\langle Y_{U}\rangle }_{2L-2}^{*}\\ \vdots & \vdots & \ddots & \vdots \\ {\langle Y_{U}\rangle }_{2L-1} & {\langle Y_{U}\rangle }_{2L-2} & \cdots & {\langle Y_{U}\rangle }_{L} \end{array}\right]. \end{array}$$

From Equation (22), we know there are some zero elements in $Y_{U}$, so several elements in $\tilde{R}$ corresponding to the interpolated virtual sensors may be zeros. In order to reconstruct covariance matrix, taking the Hermitian Toeplitz PSD property and sparse covariance matrix $\tilde{R}$ as the prior knowledge, an atomic norm minimization method can be formulated as

(39) $$\begin{array}{c} \min_{z\in C^{L}}\;{∥\begin{array}{c} z \end{array}∥}_{A_{r}}\\ \mathrm{subject}\;\mathrm{to}\;{∥\begin{array}{c} (T\left(z\right)-\tilde{R})⊙H \end{array}∥}_{F}^{2}\leq \epsilon \\ \;\;\;T(z)\geq 0 \end{array}$$

where $H={\mathrm{hh}}^{T}\in R^{L\times L}$, and $h\in R^{L}$ is a binary vector defined to represent the reference virtual array, in which the elements corresponding to interpolated virtual sensors are set to 0 and others corresponding to derived virtual sensors are defined to 1. Accordingly, H is a binary matrix to distinguish the zero and non-zero elements in covariance matrix. ϵ denotes an error threshold to restrict noise and deviation between the non-zero elements in $\tilde{R}$ and the elements corresponding to derived virtual sensors in the reconstructed covariance matrix T(z), which guarantees to reduce noise. By Equation (39), all observations received by the derived virtual sensors in difference co-array $A_{v}$ are used to denoise. Meanwhile, the zero elements corresponding to interpolated virtual sensors in $\tilde{R}$ can be recovered as

(40) T=T(z)⊙(I−H),

where I denotes the identity matrix. Using the Lagrange interpolation method, the optimization problem can be further reformulated as

(41) $$\begin{array}{c} \min_{z\in C^{L}}\;\frac{1}{2}{∥\begin{array}{c} (T\left(z\right)-\tilde{R})⊙H \end{array}∥}_{F}^{2}+\tau {∥\begin{array}{c} z \end{array}∥}_{A_{r}}\\ \mathrm{subject}\;\mathrm{to}\;T(z)\geq 0 \end{array}$$

where τ is a penalty factor to adjust the deviation and the atomic norm term. Owing to Equation (34), we found

(42) $$\mathrm{Tr}\left(T\left(z\right)\right)=L\sum_{k=1}^{K}p_{k}=L{∥\begin{array}{c} z \end{array}∥}_{A_{r}}.$$

Substitute Equation (42) into Equation (41), the optimization can be rewritten as

(43) $$\begin{array}{c} \min_{z\in C^{L}}\;\frac{1}{2}{∥\begin{array}{c} (T\left(z\right)-\tilde{R})⊙H \end{array}∥}_{F}^{2}+\lambda \mathrm{Tr}\left(T\left(z\right)\right)\\ \mathrm{subject}\;\mathrm{to}\;T(z)\geq 0 \end{array}$$

where λ=τ/L is also the penalty factor. The above optimization equation is convex, and its optimum solution $z^{*}$ can be solved by CVX software tool based on the interior point methods. And then, using $z^{*}$ as the first column, the covariance matrix $T(z^{*})$ of reference virtual array can also be reconstructed with more DOFs than the number of physical sensors. Finally, applying the traditional DOA estimation methods such as MUSIC or ESPRINT [35] to $T(z^{*})$, the DOAs of wideband LFM signals can be solved with more DOFs and better accuracy. Throughout the theoretical derivation above, the proposed method is summarized in Algorithm 1.

**Algorithm 1** Underdetermined DOA Estimation of Wideband LFM Signals Based on Gridless Sparse Reconstruction in FRF Domain.**Input:** the received signals based on the coprime array x(t); **Output:** ${\hat{\theta }}_{k}$;1:**Initialize:**M,N,L=M(N−1)+1;2:Derive the peaks model $X_{F}$ of received signals in FRF domain based on coprime array by Equation (15);3:Derive the virtual received signals $Y_{v}$ of difference co-array by Equation (21);4:Construct the virtual received signals $Y_{U}$ of virtual ULA according to Equation (22) by interpolating virtual sensors into difference co-array;5:Construct the covariance matrix of virtual ULA $\tilde{R}$ according to Equation (38);6:Defined a binary vector h to respectively represent the interpolated and derived virtual sensors, and construct H to distinguish the zero and non-zero elements in covariance matrix $\tilde{R}$;7:Solve the atomic norm minimization in Equation (39), and use the equivalent version Equation (43) to get z and T(z);8:Substitute T(z) as the recovered covariance matrix of reference virtual array into MUSIC algorithm to estimate the DOAs ${\hat{\theta }}_{k}$.

## 4. Simulation Results

In this section, the simulation results will be presented to verify the performance of the proposed method with respect to resolution, DOFs and accuracy of DOA estimation. Consider a pair of coprime arrays with M+N−1=7 isotropic physical sensors located at {0,3d,5d,6d,9d,10d,12d}, where *M* = 3 and *N* = 5. As for the wideband LFM signals, the ratio between bandwidth and the center frequency $W_{b}/f_{c}$ is equal to 2/3. The sampling frequency $f_{s}$ is three times as much as the highest frequency of the signal. The number of snapshots is P = 1024. For simplicity, assume that the power of wideband LFM signal is $\sigma_{s}^{2}$, the SNR is defined as $10log_{10}\left(\sigma_{s}^{2}/\sigma_{n}^{2}\right)$. The proposed method would be compared with the wideband DOA estimation algorithms based on incoherent signal-subspace method (ISM) [9], FRFT (FRFT-MUSIC) [16], sparse representation with $l_{p}$-norm (FRFT-MAP) [21], sparse sampling MUSIC (FRFT-CSSM) [25], sparse recovery by compressive sensing (FRFT-OMP) [27] nuclear norm sparse recovery (FRFT-NN) [28] and spatial compressive sensing using randomly thinned array (ISM-CSRTA) [22], respectively. FRFT-CSSM, FRFT-OMP and FRFT-NN methods are obtained by performing FRFT on the received signals and then using CSSM, OMP and nuclear norm algorithms to estimate DOAs, respectively. The interval of the pre-defined grids is set to $0.1^{\circ }$ for the FRFT-MAP, FRFT-OMP and ISM-CSRTA algorithms. The penalty factor λ for the proposed algorithm is set to be 0.25.

**Figure 5:**
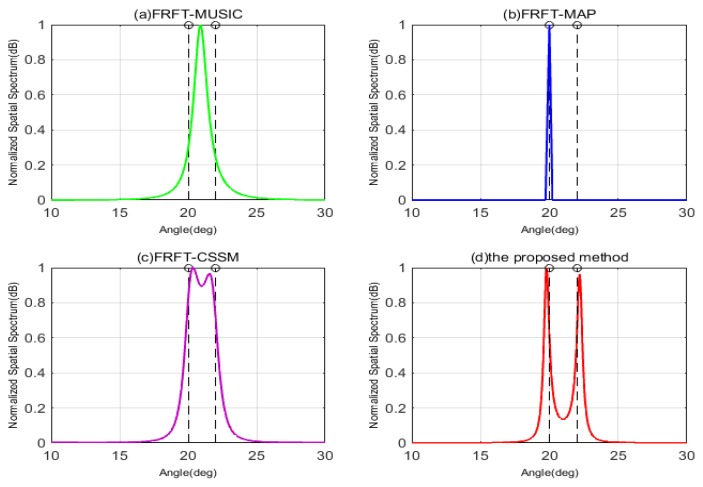
The normalized MUSIC spatial spectrums by four different algorithms with SNR = 10 dB, Δθ = $2^{\circ }$. (**a**) FRFT-MUSIC; (**b**) FRFT-MAP; (**c**) FRFT-CSSM; (**d**) The proposed method.

**Figure 6:**
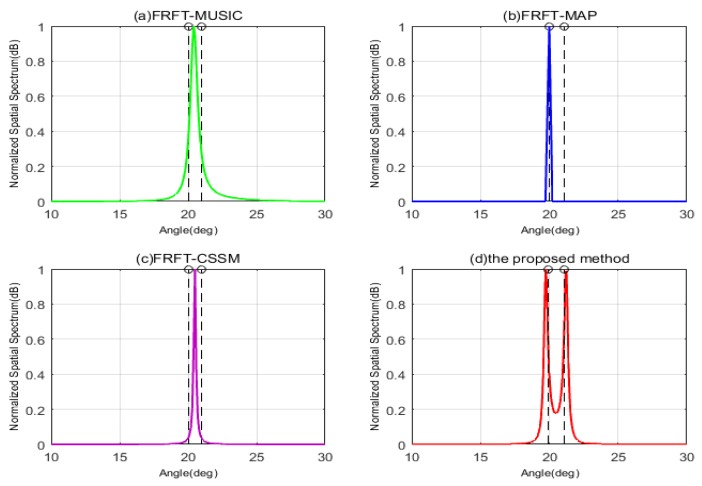
The normalized MUSIC spatial spectrums by four different algorithms with SNR = 10 dB, Δθ = $1^{\circ }$. (**a**) FRFT-MUSIC; (**b**) FRFT-MAP; (**c**) FRFT-CSSM; (**d**) The proposed method.

The first simulation takes the resolution of DOA estimation into consideration, and assumes that there are two closely spaced wideband LFM signals impinging on the coprime array with SNR = 10 dB, respectively. The first wideband signal comes from $\theta_{1}$ = $20^{\circ }$ fixedly, and the DOA of the second wideband signal is set as $\theta_{2}$ = $\theta_{1}$ + Δθ, where Δθ is the angular difference varied from $2^{\circ }$ to $1^{\circ }$. That is also to say the second LFM signal comes from $22^{\circ }$ and $21^{\circ }$, respectively. In Figure 5, when Δθ is $2^{\circ }$, it can be clearly seen that the FRFT-MUSIC and FRFT-MAP are unable to resolve the two signals, but FRFT-CSSM and the proposed method can accurately estimate DOAs of two wideband signals. That is because FRFT-MUSIC and FRFT-MAP methods are both based on ULA, which results in their resolutions constricted by the number of physical sensors in ULA. If the DOAs of two wideband signals stay too close, which exceeds their resolution abilities, these two methods cannot distinguish the DOAs of signals. FRFT-CSSM and the proposed method are based on the coprime array, and their DOFs are extended by vectoring covariance matrix, which are greater than the number of physical sensors, so their resolutions have been improved. Meanwhile, compared FRFT-CSSM with the proposed method in Figure 5c,d, we can observe that although peaks of FRFT-CSSM point at the directions of wideband signals, its spectrum is not as sharp as that of the proposed method. Theoretically speaking, this is because the FRFT-CSSM algorithm only exploits the consecutive part in the difference co-array and discards the information of the non-consecutive part, its DOF is smaller than that of the proposed method and its resolution would decline. As shown in Figure 6, when Δθ decreases to $1^{\circ }$, the FRFT-MUSIC, FRFT-MAP and FRFT-CSSM methods all fail to identify the two sources, but only the proposed method has two shape peaks at the DOAs of two closely spaced signals. This is because the proposed method not only uses all the sensors in the difference co-array, but also interpolates virtual sensors whose information is recovered by the gridless sparse reconstruction. It exhibits a more superior resolution than the other algorithms with the same number of physical sensors, owing to the DOF increase.

**Figure 7:**
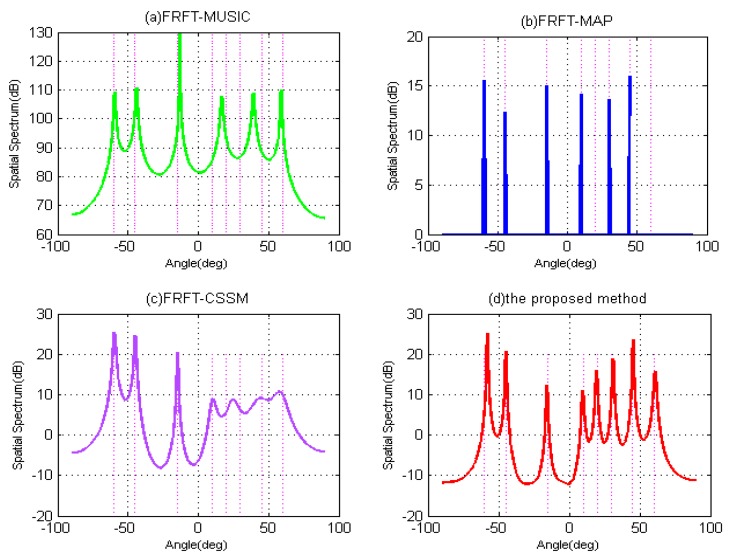
DOFs performance comparison with respect to spatial spectrum when 8 wideband LFM signals impose on the array. (**a**) FRFT-MUSIC; (**b**) FRFT-MAP; (**c**) FRFT-CSSM; (**d**) The proposed method.

In what follows, to validate the DOFs improvement by the proposed method, the second simulation is carried out, when the number of wideband LFM signals exceeds the number of physical sensors. The tested algorithms all use seven physical sensors in received array with SNR = 10 dB and P = 1024. In Figure 7, suppose that there are eight uncorrelated wideband signals arriving from different directions $\left\{-60^{\circ },-45^{\circ },-15^{\circ },10^{\circ },20^{\circ },30^{\circ },45^{\circ },60^{\circ }\right\}$ impinging on the array, which are drew as the vertical pink lines in Figure 7. As can be seen in Figure 7a–c, while the number of signals surpasses the DOFs of algorithms, the FRFT-MUSIC, FRFT-MAP and FRFT-CSSM methods only can resolved some signals, but the other peaks of spatial spectrum become flatter and mix together, which results in the loss of DOAs of some signals and the unprecise resolution. Theoretically, FRFT-MUSIC and FRFT-MAP methods are both based on ULA, and their DOFs cannot be greater than the number of physical sensors, i.e., DOF ≤ 7, which are not able to estimate 8 uncorrelated signals simultaneously. As for FRFT-CSSM, because it is based on coprime array with seven sensors, the number of available consecutive virtual sensors in the difference co-array is increased to eight. Its DOF ≤ 8, which means it can resolve seven uncorrelated signals at most. In Figure 7d, it can be clearly observed that when there are eight wideband signals impinging on the array, the proposed method has the ability to estimate their DOAs correctly at the same time. The number of available sensors is significantly increased to M(N−1)+1=13 by interpolating the virtual sensors. That is also to say the DOF of the proposed method reaches 13, and it can resolve 12 different wideband signals at most. Hence, when there are eight wideband signals impinging on the array, the other algorithms all fail, but the proposed method is valid to estimate the DOAs of eight or more wideband signals accurately.

Afterwards, the accuracy performance of DOA estimation algorithms would be assessed by the subsequent simulations in detail. The root mean square error (RMSE) of the DOA estimations is adopted as the performance index

(44) $$\mathrm{RMSE}=\sqrt{\sum_{k=1}^{K}\sum_{q=1}^{Q}\frac{{\left({\hat{\theta }}_{k}\left(q\right)-\theta_{k}\right)}^{2}}{QK}}$$

where ${\hat{\theta }}_{k}\left(q\right)$ is the estimation of $\theta_{k}$ for the *q*th Monte Carlo trial, *K* is the number of wideband LFM signals and *Q* denotes the number of Monte Carlo trials. In subsequent simulations, we run 500 Monte Carlo trials for each level. Consider the three signals impinging on the coprime array come from distinct directions $\left\{-20^{\circ },30^{\circ },60^{\circ }\right\}$, respectively.

**Figure 8:**
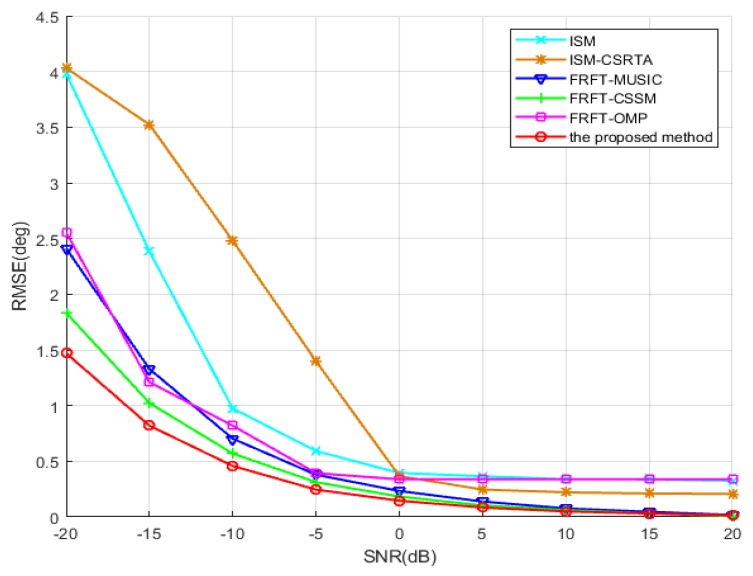
RMSE of the DOA estimations vs. input SNR.

In the third simulation, the RMSE of the DOA estimations is compared in respect of different input SNRs, which vary from −20 to 20 dB with an interval of 5 dB. The number of snapshots is 1024. As shown in Figure 8, the result illustrates that the DOA estimations by the proposed method become more and more accurate as SNR increases, and obviously outperform those by ISM, ISM-CSRTA, FRFT-MUSIC, FRFT-CSSM and FRFT-OMP approaches. Theoretically speaking, ISM-based algorithms decompose the wideband signals into several independent narrowband signals, and approximately assume that the frequency stays time-invariant in every frequency bin, without taking the continuous and linear time-varying characteristic of frequency and the whole wideband information into account. Moreover, they are based on the ULA or random array, and their DOFs are limited by the number of physical sensors. Hence, their accuracies of DOA estimation would be seriously affected. In view of a bigger aperture in the random array than that in the ULA, the RMSE of the ISM-CSRTA is slightly better than that of the ISM at high SNR. By contrast, the FRFT-based methods can estimate DOA of wideband LFM signals with time-variant frequency and steering vector, by transforming to time-invariant ones in FRF domain as shown in Equation (15). The RMSE of DOA estimation by FRFT-MUSIC is improved, but still based on the ULA, which is also constrained by the number of physical sensors. FRFT-CSSM method selects the maximum contiguous part of the difference co-array and forms an ULA, which has more sensors than FRFT-MUSIC method, so its DOF is improved to a certain extent and its accuracy of DOA estimation is better than that of FRFT-MUSIC method. Nevertheless, this method gives up the information of the discontiguous part in the difference co-array, so its accuracy performance would be compromised. Although FRFT-OMP method adopts all sensors of the difference co-array to improve DOF, it is based on the CS framework. Because the discrete grids are pre-defined at several spatial directions, it inherently has the grid mismatch problem, which would significantly degrade the accuracy of DOA estimation especially when DOAs of signals deviate from the discrete grids. Moreover, it exploits discrete basis (such as $l_{1}$ norm) to recover signal without noise, which is inevitable to bring substantial bias. In order to overcome the above shortcomings, in the proposed method, atomic norm minimization is adopted to reconstruct the covariance matrix, which has a continuous spatial parameter and effectively avoids the discrete grid mismatch problem. Moreover, the proposed method constructs a longer ULA by interpolating the virtual sensors, whose DOF is much bigger than that of the other algorithms. Therefore, the proposed method has a more accurate DOA estimation performance.

**Figure 9:**
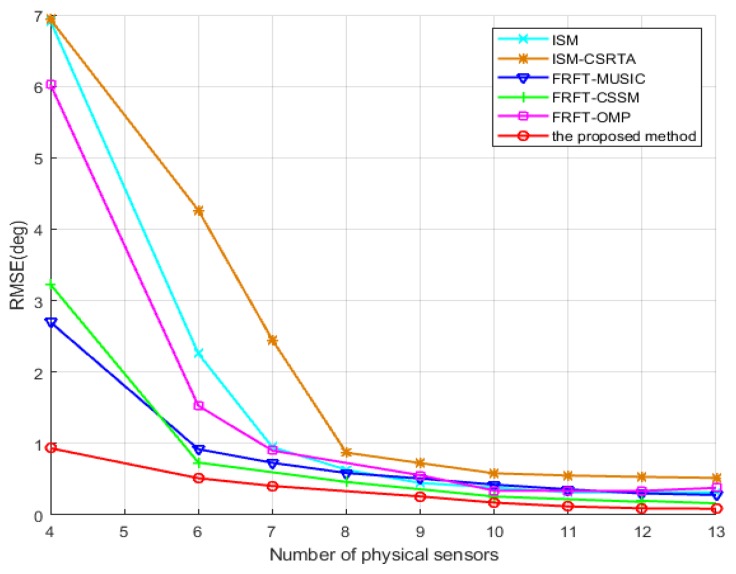
RMSE of the DOA estimations vs. number of physical sensors.

The fourth simulation further investigates the same scenario as the above one yet at a different number of physical sensors in the coprime array, which illustrates the influence of the number of physical sensors on accuracy. The number of physical sensors varies from 4 to 13 with SNR = −10 dB. As can be seen in Figure 9, the proposed method improves the estimation performance as the number of physical sensors increases, and its RMSE is much smaller than that of ISM, ISM-CSRTA, FRFT-MUSIC, FRFT-CSSM and FRFT-OMP methods, especially with less physical sensors. As analysis in FTFT-MUSIC [16], once the number of wideband sources remains the same, the smaller the number of physical sensors becomes, the bigger its RMSE is. Likewise, FRFT-CSSM employs the maximum contiguous ULA part of the difference co-array to increase available virtual sensors, but if the number of physical sensors decreases, the available virtual sensors would drastically reduce, and the RMSE would also become bigger. Although FRFT-OMP employs all the virtual sensors of the difference co-array to increase DOFs, the CS recovery approach would result in the grid mismatch and deviation from discrete grids. Furthermore, with the reduction of number of physical sensors, the number of virtual sensors in the difference co-array also declines sharply, which causes the accuracy of DOA estimation degrade severely. With the same number of physical sensors, the DOF of the proposed method is much bigger than that of the other methods. The result also shows that the accuracy of the proposed algorithm is significantly improved, which surpasses that of the other methods, especially with less sensors.

**Figure 10:**
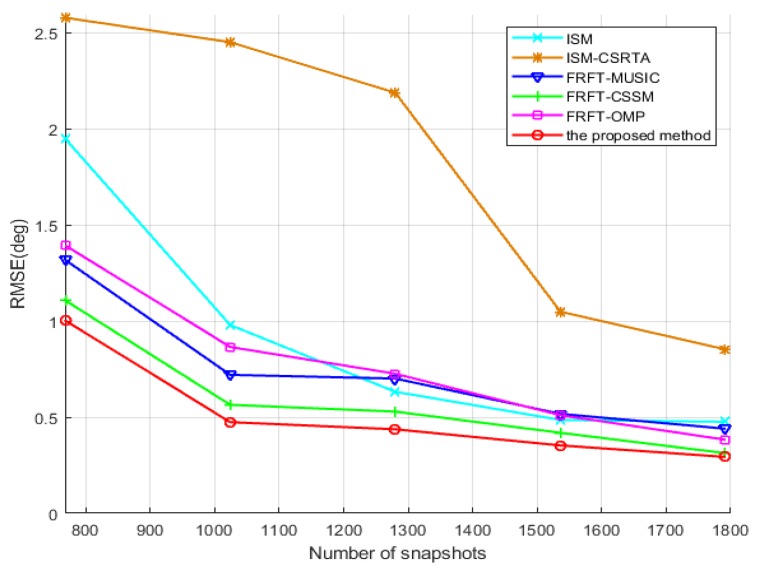
RMSE of the DOA estimations vs. the number of snapshots.

In the following, estimation accuracy of the proposed method with respect to the number of snapshots would be verified in the fifth simulation. The number of snapshots varies from 768 to 1792 with an interval of 256. The number of physical sensors is 7 in the coprime array and SNR = −10 dB. From Figure 10, the result illustrates that RMSE of the DOA estimations by the proposed method becomes smaller and smaller as the number of snapshots increases, and is superior to that of ISM, ISM-CSRTA, FRFT-MUSIC, FRFT-CSSM and FRFT-OMP approaches once again.

**Figure 11:**
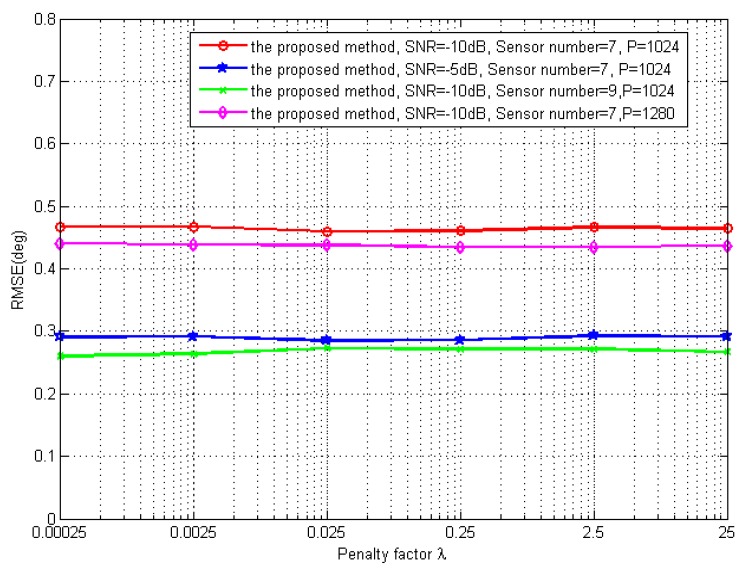
RMSE of the DOA estimations vs. the penalty factor λ.

The sixth simulation discusses the influence of the penalty factor λ. Consider the same scenario as the aforementioned ones yet at different penalty factors in optimization. In Figure 11, it is evident that the RMSE remains the same while the penalty factor λ increases from 0.00025 to 25, even with the different SNR, the number of physical sensors or the number of snapshots, respectively. The proposed method is robust with respect to the change of the penalty factor λ. That is also the reason why we choose λ = 0.25 in the simulation.

**Figure 12:**
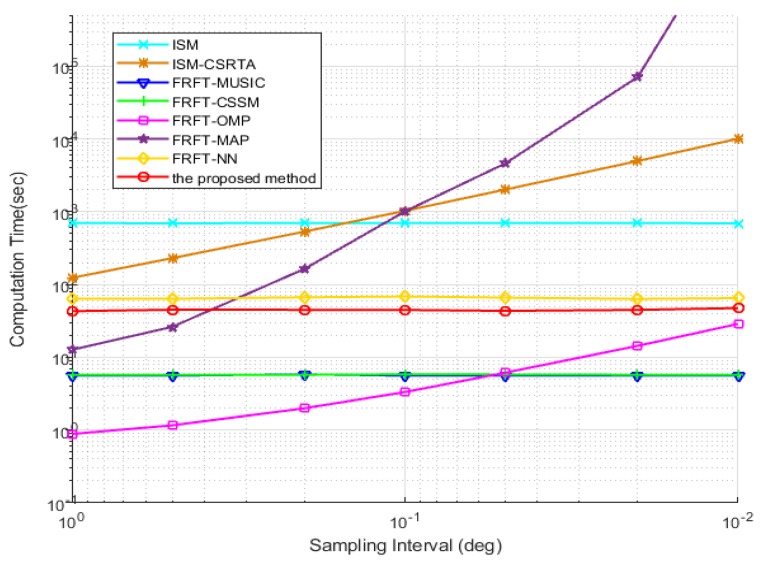
The computation time of DOA estimations vs. the sampling interval.

In the last simulation, the computational complexity measured by the computation time is verified for 100 Monte Carlo trials, which uses on a desktop with an Intel Core i7-7600U CPU. The sampling interval is varied from to $1^{\circ }$ to $0.01^{\circ }$. In Figure 12, it can be clearly observed that the computation times of the CS-based algorithms, such as ISM-CSRTA, FRFT-OMP and FRFT-MAP, increase when the grid intervals reduce and the pre-defined discrete grids become denser. However, the gridless DOA algorithms have nothing to do with the discrete grids, so they do not change with grid interval. Among them, ISM has a higher computational complexity than other gridless wideband DOA estimation algorithms, because it needs to estimate the DOA in every frequency bin and repeat this process in all frequency bins. The proposed method based on the continuous dictionary solves the optimization and recovers the sparse signals without the pre-defined discrete grids, so its computation time also has no connection with discrete grids and its sampling interval. Although it has slightly longer computation time than FRFT-MUSIC and FRFT-CSSM algorithms, it solves the underdetermined problem in wideband DOA estimation, which is insolvable by the other algorithms, and its DOF and estimation performance became better than the other wideband DOA algorithms.

## 5. Conclusions

In the proposed method, the received model of wideband LFM signals based on the coprime array is constructed in the FRF domain. In order to obtain more DOFs, by vectoring the covariance matrix and interpolating the virtual sensors, a virtual extended ULA is obtained, and its covariance matrix is recovered by using the sparse matrix reconstruction. Compared with some existing sparse reconstruction algorithms that exploit $l_{0}$ norm, $l_{1}$ norm or nuclear norm, atomic norm minimization, as a continuous dictionary without discrete grids, is used to recover the covariance matrix in order to avoid the grid mismatch problem. Moreover, unlike the existing algorithms that assume the steering vector of wideband signals is time-invariant in every frequency bin, the proposed method can resolve more wideband LFM sources than physical sensors with a time-variant steering vector in the FRF domain, but also obtain more accurate DOA estimation performance with gridless sparse reconstruction. Simulation results also demonstrate that the proposed wideband DOA estimation algorithm can to resolve the wideband LFM sources more than the number of physical sensors, and acheive more accurate DOA estimation performance.
